# Collective Narcissism and In-Group Satisfaction Predict Opposite Attitudes Toward Refugees via Attribution of Hostility

**DOI:** 10.3389/fpsyg.2019.01901

**Published:** 2019-09-04

**Authors:** Karolina Dyduch-Hazar, Blazej Mrozinski, Agnieszka Golec de Zavala

**Affiliations:** ^1^Institute of Psychology, SWPS University of Social Sciences and Humanities, Warsaw, Poland; ^2^Institute of Psychology, SWPS University of Social Sciences and Humanities, Poznań, Poland; ^3^Department of Psychology, Goldsmiths, University of London, London, United Kingdom; ^4^University Institute of Lisbon (ISCTE), Lisbon, Portugal

**Keywords:** collective narcissism, in-group satisfaction, hostile attribution bias, hostility toward Syrian refugees, refugee crisis

## Abstract

We examined *whether* and *why* collective narcissism (i.e., resentment for insufficient recognition of the in-group’s importance) versus in-group satisfaction (i.e., a belief that the in-group and one’s membership in it are reasons to be proud) have opposite, unique associations with hostility toward Syrian refugees in Poland. Results of two cross-sectional studies (Study 1, *N* = 1066 and Study 2, *N* = 419) converge to indicate that collective narcissism predicts hostility toward Syrian refugees via attributing Syrian refugees with hostile intentions toward Poles. In-group satisfaction is associated with rejection of hostile actions toward Syrian refugees because it decreases hostile attribution bias with regards to Syrian refugees. Thus, being a satisfied member of a national group promotes tolerance toward refugees, while collective narcissism is associated with blaming refugees for provoking the in-group’s hostility.

## Introduction

A majority of Poles support violence as a way of approaching the refugee crisis (Świderska et al., 2016). In the present research, we investigate why violence and hostility may seem an appropriate reaction toward people deprived of security and shelter, fleeing from persecution in their own country. We examine whether hostility toward Syrian refugees is a function of the beliefs people hold about their nation and about Syrian refugees. Opinion polls in Poland show that about three-quarters of respondents reject refugees from the Middle East perceiving them as a threat to national security (Duval Smith, 2015, October 23; Broomfield, 2016, May 9; Hall and Mikulska-Jolles, 2016; Łaciak and Segeš-Frelak, 2018), and as culturally dissimilar (Świderska et al., 2016). The rejection of refugees in Poland and a sudden decrease in openness to immigration between 2015 and 2016 has been attributed to the promotion of Polish national grandiosity after the ultra-conservative *Prawo i Sprawiedliwość* party (Law and Justice Party) came to power. However, the attitudes toward refugees in Poland vary, depending on party electorate: Voters of liberal parties such as *Razem* party (Together Party) and *Sojusz Lewicy Demokratycznej* party (Democratic Left Alliance Party) are for the settlement of refugees in Poland, whereas voters of conservative parties such as *Prawo i Sprawiedliwość* party (Law and Justice Party) and Kukiz’15 Party are against accepting refugees (Centrum Badania Opinii Społecznej [CBOS], 2017, January).

We investigate whether collective narcissism and in-group satisfaction make distinct predictions for attitudes toward Syrian refugees via hostile attribution bias. Collective narcissism is a belief that the in-group is exceptional and entitled to privileged treatment, but it is not sufficiently recognized by others (Golec de Zavala et al., 2009, 2019a; Golec de Zavala, 2011). In-group satisfaction is a belief that the in-group and one’s membership in it are reasons to be proud (Leach et al., 2008).^1^ Resentment for unrecognized greatness of the in-group is crucial to collective narcissism, whereas in-group satisfaction emphasizes pride in the in-group’s valuable features. Those two beliefs about the in-group overlap, but have strikingly different consequences for intergroup relations, especially when their common variance is partialled out (Golec de Zavala et al., 2019a). Collective narcissism without in-group satisfaction is group-based entitlement lacking the comfort of belonging to a valuable in-group, the demand for privileged treatment and the concern about loss of the in-group’s external recognition. In-group satisfaction without collective narcissism is a positive evaluation of the in-group, independent of external recognition and resilient to threats or criticism (Golec de Zavala, 2011; Golec de Zavala et al., 2013a, 2019b).

We examine whether collective narcissism and in-group satisfaction have opposite, unique relationships with hostility toward Syrian refugees. This prediction is in line with the rich literature indicating that some forms of national in-group positivity have different associations with intergroup attitudes e.g., nationalism vs. patriotism (Green et al., 2011; Wagner et al., 2012) or in-group glorification vs. in-group attachment (Roccas et al., 2008); for review see Golec de Zavala et al. (2019a) and Golec de Zavala and Schatz (2013). It is also in line with previous findings that collective narcissism and in-group satisfaction have opposite unique associations with intergroup hostility: Collective narcissism is related to intergroup hostility positively, whereas in-group satisfaction is related to intergroup hostility negatively (Golec de Zavala et al., 2019a). However, to the best of our knowledge, no previous research attempted to understand *why* those variables make unique opposite predictions for attitudes toward out-groups. We expect that they do because they are differentially linked to a tendency to attribute out-groups with hostility toward the in-group i.e., hostile attribution bias. Thus, hostile attribution bias may mediate the unique, opposite links between collective narcissism and in-group satisfaction with hostility toward refugees.

## Collective Narcissism, In-Group Satisfaction and Hostile Attribution Bias

Literature has established a reliable link between collective narcissism and intergroup hostility. A meta-analytical summary indicates a robust relationship with a small to medium effect size (Golec de Zavala et al., 2019a). Collective narcissism predicts prejudice (Lyons et al., 2010; Golec de Zavala et al., 2013a) and retaliatory hostility in response to past, present, actual and imagined offenses toward the in-group (Golec de Zavala et al., 2009, 2013a, 2016). When people hold the collective narcissistic belief about their in-group, they exaggerate their in-group’s importance and are convinced the in-group’s true worth is not sufficiently appreciated by others. The in-group’s entitlement and resentment due to the lack of the in-group’s recognition are crucial to collective narcissism. The perception of a continuous threat to the in-group’s image is inherent in the collective narcissistic belief about the in-group (Golec de Zavala et al., 2009, 2019a; Golec de Zavala, 2011). Collective narcissism is also linked to negative emotionality and sensory processing sensitivity i.e., genetically determined hypersensitivity to negative stimuli (Aron and Aron, 1997; Golec de Zavala, 2019). In addition, collective narcissism is negatively associated with social connectedness and gratitude (Golec de Zavala, 2019) but positively associated with self-criticism, low self-esteem (Golec de Zavala et al., 2019b) and vulnerable individual narcissism i.e., antagonistic self-entitlement manifesting in a distrustful and neurotic interpersonal style (Miller et al., 2017; Golec de Zavala et al., 2019a).

Those findings suggest that defensiveness, a motivation to protect the positive image of the in-group, and distrustful approach to others are characteristic of collective narcissism. When people hold the collective narcissistic belief about their in-group, they are likely to approach out-groups with suspicion and see them as harboring hostile intentions toward the in-group. This biased perception may be used to justify the in-group’s hostility toward such out-groups. In-group’s hostility can be seen as a defense in response to provocation. The hostility may differ in its expression depending on the examined national group. In this vein, research shows that the relationship between American collective narcissism and support for the military invasion in Iraq in 2003 was mediated by the perception of the national in-group as threatened by hostility of other groups (Golec de Zavala et al., 2009). Studies also showed that the link between Polish collective narcissism and anti-Semitism was mediated by the conspiracy stereotype of Jews, according to which Jews threaten Poles by their secretive intention to dominate the world (Kofta and Sędek, 2005; Golec de Zavala and Cichocka, 2012). Similarly, Americans who hold collective narcissistic belief about the United States, perceived Arabs as “wishing to harm the United States,” and held prejudice toward Arabs, but not toward other out-groups such as Asians, Europeans or Latinos whom they did not perceive as threatening (Lyons et al., 2010). Finally, suggestive of a tendency to react with hostility to perceived provocation, collective narcissism was linked to glorifying revenge against those who wrong the in-group (Dyduch-Hazar et al., in preparation). Such findings suggest that people who endorse collective narcissistic belief about the in-group may perceive themselves as obliged to retaliate and punish out-groups for being hostile toward the in-group. Thus, collective narcissism is likely to be associated with hostility toward refugees via attributing them with hostility toward the national in-group. The opposite can be expected for in-group satisfaction.

Pride and happiness of being a member of a valuable in-group is crucial to in-group satisfaction (Leach et al., 2008). In-group satisfaction is not related to intergroup hostility after the in-group image threat (Golec de Zavala et al., 2013b) or hypersensitivity to in-group criticism (Golec de Zavala et al., 2016). When the common variance shared by collective narcissism and in-group satisfaction is removed, in-group satisfaction is uniquely, negatively associated with out-group derogation (Golec de Zavala et al., 2013a, 2019b) and with a tendency to accept past transgressions of the in-group against an out-group (Dyduch-Hazar et al., in review).

In contrast to collective narcissism, in-group satisfaction is uniquely associated with positive emotionality, pro-sociality and psychological well-being (Golec de Zavala, 2019) and high self-esteem (Gramzow and Gaertner, 2005; van Veelen et al., 2011; Amiot and Aubin, 2013; Golec de Zavala et al., 2019b). Longitudinal analyses indicate that its relationship with self-esteem is reciprocal. High self-esteem predicts future in-group satisfaction and in-group satisfaction predicts future self-esteem (Golec de Zavala et al., 2019b). In addition, in-group satisfaction, is associated with the belief that the positive characteristics of individuals should be used to enhance the valuable in-group (Amiot and Sansfaçon, 2011; Jans et al., 2011; Legault and Amiot, 2014), whereas collective narcissism is not associated with a concern about the in-group’s welfare (Jaworska, 2016). Thus, unlike collective narcissism, in-group satisfaction is associated with a positive, pro-social, and tolerant approach. It is likely to be associated with willingness to help others in need, even when they belong to an out-group, and rejection of hostile attribution bias.

## Present Studies

In two cross-sectional studies, we tested the hypothesis that collective narcissism and in-group satisfaction have opposite, unique associations with hostility toward Syrian refugees via the tendency to attribute refugees with hostile intentions toward Poles (Hypothesis 1). We also expected that collective narcissism and in-group satisfaction mutually suppress each other’s relationships with attributing refugees with hostility toward Poles (Hypothesis 2). Previous studies have shown that collective narcissism and in-group satisfaction often mutually suppress each other’s opposite relationships with intergroup hostility (Golec de Zavala et al., 2013a, in preparation).

The studies were conducted in Poland, where the collective narcissistic rhetoric about the country’s threatened and misunderstood greatness has been increasingly present in public life, especially since the ultra-conservative, populist party *Prawo i Sprawiedliwość* (Law and Justice Party) came to power (Hedges, 2017). Poland is also one of the few European countries with the lowest level of support for helping Syrian refugees (Bieńkowski and Świderska, 2017).

The present studies used different measures of hostile attribution bias and hostility toward Syrian refugees to examine whether the findings generalize beyond one method of assessment. Study 1 used data from a nationwide survey based on a representative sample of Polish adults. In this study, hostility toward Syrian refugees was measured by two items pertaining to feelings toward and preferred social distance from Syrian refugees. Study 2 was a cross-sectional study, which aimed to replicate the results of Study 1 with an extended measure of hostile attribution bias and a more direct measure of hostile behavioral intentions. In this Study, we measured the extent to which participants would like to engage in hostile actions against Syrian refugees (Mackie et al., 2000).

In both studies, we applied a stepwise analytic strategy. First, we aimed to determine whether the four examined variables (collective narcissism, in-group satisfaction, hostile attribution bias, and hostility toward refugees) are distinct and their measurements corresponded to four distinct latent factors. We compared the four-factor model differentiating the four variables with an alternative model in which one latent factor represented all four variables combined and another alternative model with two latent factors - one representing collective narcissism and in-group satisfaction combined and another factor representing hostility attribution bias and hostile behavioral intentions combined.

Next, we examined whether collective narcissism had a unique, positive, indirect association with intergroup hostility via hostile attribution bias and whether in-group satisfaction, independently, had a unique, negative, indirect association with intergroup hostility via rejection of hostile attribution bias. The tested model proposes a psychological process through which the beliefs about the positive value of the in-group, which we label collective narcissism versus in-group satisfaction, are linked to hostility toward refugees via the perception of the targeted out-group as hostile toward the national in-group.

We then tested whether collective narcissism and in-group satisfaction acted as mutual suppressors of their relationships with hostile attribution bias. Suppression occurs when one variable increases the predictive validity of another variable, and when a direct and indirect (via suppressor) relationship between two variables have opposite signs (MacKinnon et al., 2000).

Finally, we tested our hypothesized model against an alternative model, in which collective narcissism and in-group satisfaction were treated as predictors of hostility toward refugees entered as a mediator and hostile attribution bias was entered as the outcome variable. We also compared the hypothesized model to an alternative model in which all relationships were reversed: Hostility toward refugees was entered as a predictor, hostile attribution bias as a mediator and collective narcissism and in-group satisfaction as outcome variables.

## Study 1

### Materials and Methods

#### Participants

Participants were 1066 Polish nationals,^2^ 500 female. The mean age was 44.39 (*SD* = 15.73). Data collection was supported by the Ariadna Research Panel^3^. All participants were 18 years old or over. After signing the informed consent, participants responded to the measures, which were presented to each participant in a different random order. The order of items was also randomized. Survey weights were applied to correctly approximate a nationally representative sample. Participants responded using a scale from 1 (*totally disagree*) to 6 (*totally agree*).

In order to estimate the required sample size to test our hypotheses, we used OpenMx software (Neale et al., 2016) to run a Monte Carlo simulation (Schoemann et al., 2014). We conservatively assumed a small effect size for the association between in-group satisfaction and hostile attribution bias, *r*a = 0.10, and the association between hostile attribution bias and hostility toward refugees, *r*b = 0.10. We approximated the effect size of the association between collective narcissism and hostile attribution bias based on the previous results regarding the relationship between collective narcissism and the conspiracy stereotype of Jews (Golec de Zavala and Cichocka, 2012), *r*a = 0.43. The effect size for the association between collective narcissism and hostility toward refugees was *r*c = 0.20 based on a meta-analytical summary (Golec de Zavala et al., 2019a). We assumed that the effect size for the relationship between in-group satisfaction and hostility toward refugees would be the same as the relationship between collective narcissism and in-group satisfaction based on previous research indicating that zero order correlations of both variables with intergroup hostility are about the same size (Golec de Zavala et al., 2013b). In order to make the results of our simulation generalizable, we replaced fixed parameter values with normal and uniform distributions.^4^ The smallest estimated sample size to discover the hypothesized indirect effect was *N* = 155 (power = 0.80, 95%CI[0.78; 0.86]), and the optimal sample size was *N* = 190 (power = 0.86, 95%CI[0.81; 0.90]). Thus, we concluded that the sample in Study 1 was sufficient to test our hypotheses.

#### Measures

##### Collective narcissism

Collective narcissism was measured by the 5-item Collective Narcissism Scale (Golec de Zavala et al., 2009; e.g., “My group deserves special treatment”), α = 0.91, *M* = 3.69, *SD* = 1.27.

##### In-group satisfaction

In-group satisfaction was measured by the 4-item in-group satisfaction subscale of the In-group Identity Scale (Leach et al., 2008; e.g., “I am glad to be Polish”), α = 0.93, *M* = 4.42, *SD* = 1.07.

##### Hostile attribution bias

Hostile attribution bias was measured by 4 items prepared for the present study: “Syrian refugees threaten our national security”; “Syrian refugees are hostile toward Poles”; “Syrian refugees are aggressive,” and “Syrian refugees are dangerous,” α = 0.91, *M* = 3.71, *SD* = 1.36.

##### Hostility toward refugees

Hostility toward refugees was measured by two items based on two well-validated measures of out-group derogation i.e., Feeling Thermometer and Social Distance measure (Bogardus, 1925): “I have warm feelings toward Syrian refugees”; “I would have nothing against a member of my family marrying a Syrian refugee.” The items were reversed so the higher scores indicate higher rejection of Syrian refugees, α = 0.80, *M* = 3.88, *SD* = 1.40.

Table 1 presents all the inter-correlations between the constructs.

**TABLE 1:** Correlation coefficients among constructs in Studies 1 and 2.

	**CN**	**IS**	**HAB**	**HTR**	**Alpha**
**Study 1**					
**(*N* = 1066)**					
Collective narcissism (CN)		0.74^∗∗∗^	0.54^∗∗∗^	0.27^∗∗∗^	0.91
In-group satisfaction (IS)	0.69^∗∗∗^		0.29^∗∗∗^	0.13^∗∗^	0.93
Hostile attribution bias (HAB)	0.49^∗∗∗^	0.27^∗∗∗^		0.68^∗∗∗^	0.91
Hostility toward refugees (HTR)	0.23^∗∗∗^	0.11^∗∗^	0.58^∗∗∗^		0.80
**Study 2**					
**(*N* = 419)**					
Collective narcissism (CN)		0.71^∗∗∗^	0.43^∗∗∗^	0.2^∗∗^	0.91
In-group satisfaction (IS)	0.65^∗∗∗^		0.1	–0.09	0.93
Hostile attribution bias (HAB)	0.39^∗∗∗^	0.1		0.46^∗∗∗^	0.90
Hostility toward refugees (HTR)	0.18^∗∗^	–0.08	0.41^∗∗∗^		0.87

*^∗∗^*p* < 0.01; ^∗∗∗^*p* < 0.001. Scale intercorrelations corrected for attenuation; Raw correlations below the diagonal; Corrected correlations above the diagonal. CN, collective narcissism; IS, in-group satisfaction; HAB, hostile attribution bias; HTR, hostility toward refugees.*

### Results

Data analysis was carried out with R 3.6.0 (R Core Team, 2018). First, we checked for normality deviations following recommendation by Cohen et al. (2003). None of the variables violate univariate normality assumptions (see Supplementary Table S1). We used a lavaan 0.6-3 package (Rosseel, 2012) to fit the hypothesized model with maximum likelihood parameter estimates and standard errors and a chi-square test statistic (MLR) robust to non-normality and non-independence of observations (Yuan and Bentler, 2000).

First, we estimated how well the hypothesized four factor model fit the data in comparison to the alternative one-factor and two-factor models. The hypothesized four factor model fit the data very well (Table 2) and significantly better than a single-factor solution (Δχ^2^(4) = 4772.6, *p* < 0.001), or a two-factor solution (Δχ^2^(5) = 1699.2, *p* < 0.001). These results suggest that collective narcissism and in-group satisfaction are distinct beliefs about the in-group and hostile attribution bias can be distinguished from hostility toward refugees (Table 2).

**TABLE 2:** Comparison of fit indices between three models for Studies 1 and 2.

***N* factors**	***N* obs**	***N* par**	**ChiSq**	**df**	***p*-value**	**CFI**	**RMSEA**	**Low. RMSEA**	**Up. RMSEA**	**SRMR**
**Study 1**										
**(*N* = 1066)**										
4	1066	36	403.88	84	<0.001	0.97	0.06	0.06	0.07	0.04
2	1066	31	2103.08	89	<0.001	0.84	0.15	0.14	0.15	0.09
1	1066	30	5176.50	90	<0.001	0.59	0.23	0.22	0.24	0.18
**Study 2**										
**(*N* = 419)**										
4	419	50	508.48	203	<0.001	0.96	0.06	0.05	0.07	0.07
2	419	45	2946.61	208	<0.001	0.63	0.18	0.17	0.18	0.18
1	419	44	5034.13	209	<0.001	0.35	0.23	0.23	0.24	0.24

All indicators showed significant positive factor loadings with standardized coefficients ranging from β = 0.78 to β = 0.91 (Supplementary Table S2). The correlations among all four latent factors were positive and significant (Supplementary Table S4).

In order to test Hypothesis 1, that collective narcissism is positively associated with hostility toward refugees via hostile attribution bias, whereas in-group satisfaction is negatively associated with hostility toward refugees via rejection of hostile attribution bias, we extended the four latent factor structure into a structural equation model. Within this model, we constrained the latent factors to have a mean of 0 and a variance of 1 (i.e., standardized them), and used bootstrapped standard errors (in order to generate bias corrected confidence intervals for indirect effects).

**FIGURE 1:**
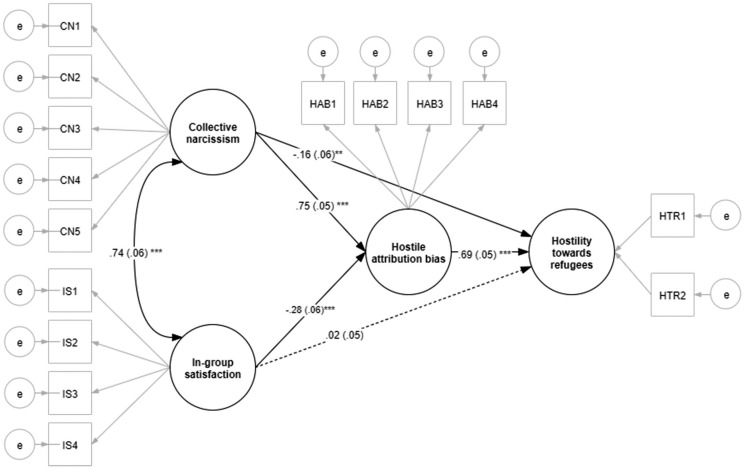
SEM diagram with standardized regression coefficients, Study 1 (*N* = 1066).

As can be seen in Table 3 and Figure 1, in line with our hypothesis the path (a1) between collective narcissism (X1) and hostile attribution bias (M) was positive and significant. As predicted, the path (b) between hostile attribution bias (M) and hostility toward refugees (Y) was also positive and significant. The indirect effect of collective narcissism on hostility toward refugees (a1^∗^b) was positive and significant. Also as expected, the path (a2) between in-group satisfaction (X2) and hostile attribution bias was negative and significant. Notably, after the overlap between in-group satisfaction and collective narcissism was partialled out, the direct association between in-group satisfaction and hostile attribution bias changed the sign and became negative. Such pattern of results suggests suppression. The indirect effect (a2^∗^b) of in-group satisfaction on hostility toward refugees via the hostile attribution bias was negative and significant. The direct effect of (c1′) was negative and significant. The direct effect (c2′) of in-group satisfaction on hostility toward refugees was not significant.

**TABLE 3:** Standardized model parameters, Study 1 (*N* = 1066).

**lhs**	**op**	**rhs**	**Beta**	**SE**	***Z***	***p*-value**
***Regressions***						
HTR	∼	CN	–0.16	0.06	–2.74	0.006
HTR	∼	IS	0.02	0.05	0.38	0.692
HAB	∼	CN	0.72	0.05	14.61	< 0.001
HAB	∼	IS	–0.28	0.06	–4.92	< 0.001
HTR	∼	HAB	0.69	0.05	15	< 0.001
***Mediation effects***						
Direct_CN	:=	c1	–0.16	0.06	–2.74	0.006
Indirect_CN	:=	a1^∗^b	0.52	0.05	10.25	< 0.001
Total_CN	:=	c1 + (a1^∗^b)	0.36	0.06	6.42	< 0.001
Direct_IS	:=	c2	0.02	0.05	0.38	0.703
Indirect_IS	:=	a2^∗^b	–0.19	0.04	–4.65	< 0.001
Total_IS	:=	c2 + (a2^∗^b)	–0.17	0.06	–2.81	0.005

*CN, collective narcissism; IS, in-group satisfaction; HAB, hostile attribution bias; HTR, hostility toward refugees.*

Finally, in order to strengthen our argument regarding the directionality of the hypothesized relationships, we tested two models in which: (1) collective narcissism and in-group satisfaction predicted hostility toward refugees, which led to hostile attribution bias, and (2) a model with reversed relationships of causality between the variables. In the first alternative model (Supplementary Table S5) the path (a1) between collective narcissism (X1) and hostility toward refugees (M) was positive and significant. The path (b) between hostility against refugees (M) and hostile attribution bias (Y) was also positive and significant. The indirect effect of collective narcissism on hostile attribution bias via hostility toward refugees was significant and positive. In comparison to our hypothesized model, this effect was visibly weaker. For in-group satisfaction as a predictor, the path (a2) predicting hostility toward refugees and the path (c2) predicting hostile attribution bias were negative and significant. The indirect effect of in-group satisfaction on hostile attribution bias via hostility toward refugees was negative and significant. This effect was also smaller than the effect in our hypothesized model. Most importantly, hostility toward refugees as a mediator left unexplained the relationship between collective narcissism and hostile attribution bias and in-group satisfaction and hostile attribution bias.

In the second alternative model (Supplementary Table S6), hostility toward refugees predicted hostile attribution bias positively (a1). Hostile attribution bias predicted collective narcissism (b1) and in-group satisfaction (b2) positively. Hostility toward refugees, predicted collective narcissism (c1) and in-group satisfaction (c2) negatively. Indirect effects predicting collective narcissism (a1^∗^b1) and in-group satisfaction (a1^∗^b2) were both positive and significant. In comparison to our hypothesized model, this alternative model did not reveal the opposite, unique associations collective narcissism and in-group satisfaction have with intergroup hostility and hostile attribution bias.

In order to test Hypothesis 2, that collective narcissism and in-group satisfaction suppress each other’s relationships with hostile attribution bias we tested two suppression effects: In-group satisfaction on the relationship between collective narcissism and hostile attribution bias and collective narcissism on the relationship between in-group satisfaction and hostile attribution bias. We expected that after the positive overlap between collective narcissism and in-group satisfaction is partialled out, the positive relationship between collective narcissism and hostile attribution bias would become stronger, whereas the association between in-group satisfaction and hostile attribution bias would change the sign and became negative and statistically significant.

As presented in Table 4, the direct effect of collective narcissism on hostile attribution bias was positive, significant and stronger than the zero-order correlation while the indirect effect via in-group satisfaction was negative and significant. The direct and indirect effect had opposite signs indicating suppression by in-group satisfaction. The direct effect of in-group satisfaction on hostile attribution bias became negative and significant while the indirect effect via collective narcissism was positive and significant, indicating suppression by collective narcissism.

**TABLE 4:** Summary of suppression effects for Studies 1 and 2.

**lhs**	**op**	**rhs**	**Beta**	**SE**	***Z***	***p*-value**
**Study 1**						
**(*N* = 1066)**						
**In-group satisfaction as suppressor**						
Direct	:=	c	0.72	0.05	14.59	< 0.001
Indirect	:=	a^∗^b	–0.18	0.04	–4.64	< 0.001
Total	:=	c1 + (a1^∗^b)	0.54	0.04	15.97	< 0.001
**Collective narcissism as suppressor**						
Direct	:=	c	–0.24	0.06	–4.86	< 0.001
Indirect	:=	a^∗^b	0.53	0.05	12.95	< 0.001
Total	:=	c1 + (a1^∗^b)	0.29	0.04	7.85	< 0.001
**Study 2**						
**(*N* = 419)**						
**In-group satisfaction as suppressor**						
Direct	:=	c	0.67	0.09	8.91	< 0.001
Indirect	:=	a^∗^b	–0.26	0.07	–4.42	< 0.001
Total	:=	c1 + (a1^∗^b)	0.41	0.06	7.90	< 0.001
**Collective narcissism as suppressor**						
Direct	:=	C	–0.37	0.10	–4.69	< 0.001
Indirect	:=	a^∗^b	0.47	0.08	7.26	< 0.001
Total	:=	c1 + (a1^∗^b)	0.09	0.07	1.54	0.123

In summary, our results suggest that collective narcissism and in-group satisfaction are distinct variables, pertaining to alternative beliefs about the in-group. A tendency to attribute the out-group with hostility is different from being hostile toward that out-group. The results of Study 1 support Hypothesis 1 indicating that collective narcissism and in-group satisfaction have opposite, unique associations with hostility toward Syrian refugees via attributing refugees with hostility toward Poles. Models assuming alternative directionality of the relationships between tested variables indicate weaker relationships difficult to interpret theoretically or did not explain the association between a predictor and the outcome. The results of Study 1 are also in line with Hypothesis 2 indicating that the positive overlap between collective narcissism and in-group satisfaction obscured the opposite associations of those variables with hostile attribution bias. In Study 2, we aimed to replicate the results of Study 1 extending our measurement of hostile attribution bias and using a more direct measurement of hostility toward refugees, i.e., the assessment of hostile behavioral intentions.

## Study 2

### Materials and Methods

#### Participants

Participants were 419 Polish nationals, 146 women. The mean age was 41.23 (*SD* = 14.14). The study was conducted online by the Ariadna Research Panel. Participants who took part in Study 1 could not take part in Study 2. The sample size was estimated like in Study 1. All participants were 18 years old or over. After signing the informed consent, participants responded to the measures, which were presented to each participant in a different random order. The order of items was also randomized. Unless otherwise indicated participants responded using the scale from 1 (*definitely disagree*) to 6 (*definitely agree*).

#### Measures

##### Collective narcissism

Collective narcissism (Golec de Zavala et al., 2009) was assessed as per Study 1, α = 0.91, *M* = 3.61; *SD* = 1.12.

##### In-group satisfaction

In-group satisfaction (Leach et al., 2008) was measured as per Study 1, α = 0.93, *M* = 4.48; *SD* = 1.03.

##### Hostile attribution bias

Hostile attribution bias was assessed by 5 items prepared for the study: Syrian refugees are: “a threat to Polish national security”; “hostile toward Polish”; “aggressive”; “dangerous,” and “helpless” (reversed), α = 0.90, *M* = 3.71, *SD* = 1.11.

##### Hostility toward refugees

Hostility toward refugees was measured by 8 items (based on Mackie et al., 2000). Participants were asked to indicate to what extent they would like to engage in each of the following behaviors toward Syrian refugees: “confront,” “oppose,” “hurt,” “humiliate,” “intimidate,” “injure,” “offend them,” and “leave them to their fate,” α = 0.87, *M* = 2.36, *SD* = 1.07.

### Results

Following the same procedure as in Study 1, we first checked whether the univariate normality assumptions were met (Supplementary Table S1). In order to fit the hypothesized four factor model, we used robust maximum likelihood estimation. The hypothesized model fit the data very well and significantly better than single-factor (Δχ^2^(6) = 4525.3, *p* < 0.001), or a two-factor solution (Δχ^2^(5) = 2438.1, *p* < 0.001, Table 2). All indicators showed significant positive factor loadings, with standardized coefficients ranging from β = 0.33 to β = 0.96 (Supplementary Table S3).

In order to test Hypothesis 1, we extended the four latent factor structure into a structural equation model, replacing covariation paths with one sided regression paths. Within this model, we constrained the latent factors to have a mean of 0 and a variance of 1 (i.e., standardized them) with bootstrapped standard errors.

**FIGURE 2:**
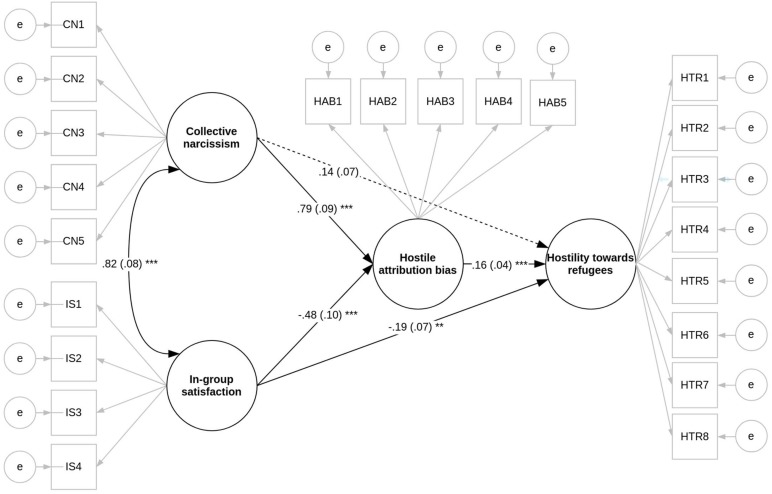
SEM diagram with standardized regression coefficients, Study 2 (*N* = 419).

As can be seen in Table 5 and Figure 2, in line with Hypothesis 1, the path (a1) between collective narcissism (X1) and hostile attribution bias (M) and the path (b) between hostile attribution bias (M) and hostility toward refugees (Y) were positive and significant. The indirect effect (a1^∗^b) of collective narcissism (X1) on hostility toward refugees (Y) was positive and significant. Unlike in Study 1, the direct effect (c1′) was non-significant. Also as expected the path (a2) between in-group satisfaction (X2) and hostile attribution bias was negative and significant. The indirect effect (a2^∗^b) of in-group satisfaction on hostility toward refugees via hostile attribution bias was negative and significant. Unlike in Study 1, the direct effect (c2′) of in-group satisfaction on hostility toward refugees was negative and significant.

**TABLE 5:** Standardized model parameters, Study 2 (*N* = 419).

**lhs**	**op**	**rhs**	**Beta**	**SE**	***Z***	***p*-value**
**Regressions**						
HTR	∼	CN	0.14	0.07	1.87	0.062
HTR	∼	IS	–0.19	0.07	–2.67	0.007
HAB	∼	CN	0.79	0.09	8.74	< 0.001
HAB	∼	IS	0.48	0.10	4.86	< 0.001
HTR	∼	HAB	0.16	0.04	4.14	< 0.001
**Mediation effects**						
Direct_CN	:=	c1	0.14	0.07	1.87	0.062
Indirect_CN	:=	a1^∗^b	0.13	0.03	4.25	< 0.001
Total_CN	:=	c1 + (a1^∗^b)	0.26	0.07	4.04	< 0.001
Direct_IS	:=	c2	–0.19	0.07	–2.67	0.007
Indirect_IS	:=	a2^∗^b	–0.08	0.02	–3.69	< 0.001
Total_IS	:=	c2 + (a2^∗^b)	–0.27	0.07	–3.74	< 0.001

*CN, collective narcissism; IS, in-group satisfaction; HAB, hostile attribution bias; HTR, hostility toward refugees.*

Following a similar analytical approach as in Study 1, we tested models assuming alternative directionality of the relationships between the variables. In the first alternative model (Supplementary Table S5), the path (a1) between collective narcissism (X1) and hostility toward refugees (M) was positive and significant. The path (b) between hostility toward refugees (M) and hostile attribution bias (Y) was also positive and significant. The indirect effect of collective narcissism on hostile attribution bias via hostility toward refugees was significant and positive but weaker than in our hypothesized model. For in-group satisfaction as predictor, the path (a2) predicting hostility toward refugees and the path (c2) predicting hostile attribution bias were negative and significant. The indirect effect of in-group satisfaction on hostile attribution bias, via hostility toward refugees, was negative and significant and stronger than in the hypothesized model. This pattern of results is consistent with the hypothesized model.

In the second alternative model (Supplementary Table S6), hostility toward refugees predicted hostile attribution bias positively (a1). Hostile attribution bias predicted collective narcissism (b1) and in-group satisfaction (b2) positively. Hostility toward refugees predicted in-group satisfaction (c2) negatively and the effect on collective narcissism (c1) was not significant. Indirect effects predicting collective narcissism (a1^∗^b1) and in-group satisfaction (a1^∗^b2) were both positive and significant. This pattern of results was not entirely consistent with the pattern obtained in Study 1. It also did not reveal the opposite associations between collective narcissism, in-group satisfaction and hostile attribution bias.

With regard to Hypothesis 2, the direct effect of collective narcissism on hostile attribution bias was positive and significant, whereas the indirect effect via in-group satisfaction was negative and significant indicating suppression. The direct effect of in-group satisfaction on hostile attribution bias was negative and significant, whereas the indirect effect via collective narcissism was positive and significant also indicating suppression (Table 4).

In summary, the results of Study 2 showed the same pattern as the results of Study 1 using different assessment of the mediator and the outcome. The results are in line with Hypothesis 1 indicating that collective narcissism and in-group satisfaction have opposite, unique indirect associations with hostile intentions toward refugees via hostile attribution bias. When extended assessments of hostile attribution bias and hostility toward refugees were applied, the direct effect of collective narcissism was not significant but the hypothesized indirect effects were replicated. Collective narcissism predicted support for hostile actions against Syrian refugees because it was positively associated with attributing refugees with hostile intentions toward Poles. In-group satisfaction was, in turn, related to the rejection of hostile actions toward Syrian refugees because it was negatively associated with the perception of refugees as hostile and dangerous. In addition, in line with Hypothesis 2, collective narcissism and in-group satisfaction acted as mutual suppressors of each other’s relationships with hostile attribution bias. The results testing alternative directionality of the relationships between variables were not consistent with those obtained in Study 1 in case of reversed causality.

## Discussion

We tested whether beliefs about a nation and Syrian refugees predicted hostility toward refugees in Poland. Results from two large, cross-sectional studies consistently indicate that collective narcissism is positively, whereas in-group satisfaction is negatively associated with hostility toward Syrian refugees via attributing Syrian refugees with hostility toward Poles. Results of a structural model analysis confirmed additionally that collective narcissism and in-group satisfaction correspond to distinct, latent factors and so do beliefs about Syrian refugees and hostility toward them. Collective narcissism was positively, whereas in-group satisfaction was negatively associated with a tendency to perceive Syrian refugees as dangerous and hostile toward Poles. Those opposite indirect associations could only be observed after the positive overlap between collective narcissism and in-group satisfaction was partialled out.

Those effects could only be observed when collective narcissism and in-group satisfaction were entered into the analyses as predictors of hostile attribution bias but not in models testing reverse causality. In addition, entering hostile attribution bias as a mediator explained the relationship between collective narcissism, in-group satisfaction and hostility toward refugees. However, the alternative model analyzing hostility toward refugees as a mediator did not explain the relationships between collective narcissism, in-group satisfaction and hostile attribution bias. Such results strengthen the evidence for the assumed directionality of the relationships between the variables.

The present results are in line with previous findings indicating that collective narcissism is a robust predictor of intergroup hostility, especially in the context of intergroup threat (Golec de Zavala et al., 2019a). They also corroborate previous findings suggesting that collective narcissism is associated with a biased perception of intergroup reality. Collective narcissism is associated with hypersensitivity to signs of threat to the in-group’s image (Golec de Zavala et al., 2016) and a tendency to see the in-group as constantly threatened by the hostility of others (Golec de Zavala et al., 2009; Golec de Zavala and Cichocka, 2012) and to a belief that the in-group must continuously defend itself against secret, hostile plots of out-groups (Golec de Zavala and Cichocka, 2012; Cichocka et al., 2016; Golec de Zavala and Federico, 2018). Going beyond such findings, the present results suggests that when people endorse the collective narcissistic belief, they see their in-group’s hostility toward out-groups as justified and defensive. They interpret their in-group’s violent actions as protection of the position of the in-group against external hostility.

In line with this interpretation, previous findings indicate that collective narcissism is associated with retaliatory intergroup aggression (Golec de Zavala et al., 2013b), which manifests itself not only as hostile behavioral intentions against out-group member (Golec de Zavala et al., 2013b) or symbolic intergroup aggression (Dyduch-Hazar et al., in preparation; Golec de Zavala et al., 2019b), but also as intergroup *schadenfreude*: rejoicing in suffering of others (Golec de Zavala et al., 2016), negative attitudes toward government and policies of the out-group (Cai and Gries, 2013) or destructive actions against companies owned by members of the out-group (Golec de Zavala et al., 2009). Such findings parallel, on the intergroup level, the results indicating that individual narcissism is associated with retaliatory interpersonal aggression (Baumeister et al., 1996), especially under a threat to the self-image (Bushman and Baumeister, 1998; Konrath et al., 2006). Collective narcissism is a belief about the in-group. However, it is associated with individual narcissism. More specifically, it is reliably associated with vulnerable narcissism i.e., distrustful resentment for the lack of personal recognition. Sometimes but less systematically, collective narcissism is associated with grandiose narcissism i.e., agentic superiority over others (Golec de Zavala, 2018; Golec de Zavala et al., 2019a). The present results suggest that just like individual narcissists, who react aggressively to protect their inflated egos (Spector, 2011; Chester and DeWall, 2016), when people endorse the collective narcissistic belief about the in-group, they interpret the actions of other groups as hostile to the in-group and lash out against them.

In contrast, when people believe their in-group is of a high value without lamenting over its unrecognized exceptionality, they do not perceive refugees as dangerous and they are not hostile toward refugees. These findings corroborate previous results indicating that in-group satisfaction facilitates intergroup generosity and co-operation (Golec de Zavala et al., 2013a, 2019b; Jaworska, 2016) and motivate in-group members to use their individual strengths to improve their in-groups (Jans et al., 2011; Legault and Amiot, 2014).

Our results support Hypothesis 2. They show that collective narcissism and in-group satisfaction suppress each other’s opposite associations with hostile attribution bias and hostility toward refugees. These results are consistent with previous findings indicating that collective narcissism and in-group satisfaction act as mutual suppressors of each other’s opposite relationships with variables pertaining to intergroup attitudes (Golec de Zavala et al., 2013a) as well as personal characteristics such as a sense of control (Cichocka et al., 2017), self-esteem (Golec de Zavala et al., 2019b), life-satisfaction or pro-sociality (Golec de Zavala, 2019). In this vein, as indicated above, studies showed that collective narcissism and in-group satisfaction predicted opposite attitudes toward out-groups (Golec de Zavala et al., 2013a) and toward accepting past transgressions of the in-group against an out-group (Dyduch-Hazar et al., in review). They also had opposite, unique relationships with the belief in rewarding power of intergroup revenge: Collective narcissism was positively, whereas in-group satisfaction was negatively associated with the belief that revenge is desirable (Dyduch-Hazar et al., in preparation). The present results expand such findings by indicating that collective narcissism and in-group satisfaction are linked to different perceptions of intergroup situations and the intentions of out-groups, which explains their opposite associations with intergroup hostility.

Our findings have at least two important implications for studies of intergroup relations. First, they highlight the importance of a belief about the in-group for harmonious intergroup relations. Collective narcissism, although a positive belief about the in-group, may become a serious obstacle for out-group tolerance (Golec de Zavala et al., 2013a, b, 2019a) and intergroup reconciliation (Dyduch-Hazar et al., in review). Second, the present results indicate that as long as in-group satisfaction is related to collective narcissism, the relationship between collective narcissism and hostile attribution bias, and therefore intergroup hostility, is reduced. When people who hold the collective narcissistic belief about the in-group are also satisfied and proud members of their in-group, they perceive out-group members as dangerous and harboring hostile intentions against their in-group to a lesser extent. This suggests that emphasizing the satisfaction of being a member of a valuable in-group may decrease collective narcissistic bias and intergroup hostility. However, the present results also indicate that as long as in-group satisfaction is related to collective narcissism, its negative relationship with hostile attribution bias is diminished. Thus, the overlap with collective narcissism may increase hostile tendencies of satisfied in-group members. Policies should, therefore, focus on strengthening in-group satisfaction (which decreases intergroup hostility associated with collective narcissism), rather than collective narcissism (which decreases out-group tolerance associated with in-group satisfaction). Collective narcissism may be strengthened by situations that may motivate to compensate for the lack of self-esteem and personal control such as economic crisis (Marchlewska et al., 2017; Golec de Zavala et al., 2019a) or social exclusion (Golec de Zavala et al., 2019a). In-group satisfaction may be strengthened by emphasizing pride to be a member of a valuable in-group and a willingness to work for the in-group’s welfare (Golec de Zavala et al., 2019a).

## Limitations

Although the present results provide important insights into the nature of the associations between collective narcissism, in-group satisfaction and hostility toward refugees, there are limitations that need to be taken into account when interpreting them. Present studies are correlational and they do not allow for firm conclusions regarding the directionality of the observed effects. We provided rationale for why broader variables pertaining to beliefs about the in-group’s positive value should determine more specific perceptions such as hostile attribution bias and specific attitudes and behavioral intentions pertaining to hostility toward refugees. We also tested our hypothesized model against the model assuming that intergroup hostility mediated the relationships between collective narcissism, in-group satisfaction and hostile attribution bias. Although we found significant indirect effects, direct effects remained significant as well. In contrast, hostile attribution bias as a mediator reduced the direct associations between collective narcissism and hostility toward refugees in Study 2 and reversed it in Study 1. Testing the second alternative model reversing the directionality of the relationships brought about results that were not consistent across the two studies. Thus, we concluded that our results support our hypothesized model. Nevertheless, future studies would do well testing this model in longitudinal and experimental designs that allow for firmer conclusions about directionality.

The present results are inconsistent regarding the direct relationship between collective narcissism and hostility toward refugees. In Study 2, this relationship remains positive but it becomes marginally significant after hostile attribution bias is entered as a mediator. In Study 1, this relationship changes a sign and becomes negative and significant. This negative association between collective narcissism and intergroup hostility has not been observed previously (Golec de Zavala et al., 2019a). Thus, this finding is difficult to interpret.

In present studies, we assessed self-reported hostility toward refugees assessed as a feeling thermometer, preferred social distance and hostile behavioral intentions. We did not measure actual behaviors toward refugees. This limits the generalizability of our findings to behavioral outcomes. However, our previous studies indicate that collective narcissism is associated not only with self-reported hostile attitudes but also with aggressive behaviors toward out-groups (Golec de Zavala et al., 2013b, 2019b; Dyduch-Hazar et al., in preparation). This increases our confidence that the present results can generalize to real-life behaviors. However, in the future it would be beneficial to measure hostility toward refugees using behavioral indicators of aggression such as noise blasts (Chester and Lasko, 2018) or the voodoo doll task (Chester and DeWall, 2017).

## Data Availability

All datasets generated for this study are included in the manuscript and/or the Supplementary Files.

## Ethics Statement

The studies presented in this manuscript were carried out in accordance with the recommendations of the British Psychological Society with written informed consent from all participants. The studies were approved by the Ethics Committee at the Department of Psychology, Goldsmiths, University of London. Studies did not involve vulnerable populations. Participants consented to take part in the studies after reading the consent form.

## Author Contributions

KD-H wrote the theoretical analysis presented in this manuscript. BM analyzed the data and presented the results. AGZ formulated the research hypotheses, developed the theoretical argument and interpretation of findings, and supervised the preparation of the manuscript.

## Conflict of Interest Statement

The authors declare that the research was conducted in the absence of any commercial or financial relationships that could be construed as a potential conflict of interest.

## Footnotes

1 The differentiation between collective narcissism vs. in-group satisfaction aligns with the literature that distinguishes between two forms of positive in-group evaluation differentially associated with out-group derogation. These two forms have been named differently (e.g., genuine patriotism and pseudo-patriotism (Adorno et al., 1950); patriotism vs. nationalism (Kosterman and Feshbach, 1989); constructive vs. blind patriotism (Schatz et al., 1999); in-group attachment vs. in-group glorification (Roccas et al., 2006) or secure vs. insecure in-group attachment (Jackson and Smith, 1999). Unlike the other concepts, collective narcissism and in-group satisfaction pertain specifically to beliefs about the in-group’s positive value and can be applied to any in-group not only the national one. The concept of collective narcissism provides an explanation of the mechanism linking in-group positivity to out-group derogation, whereas other concepts (e.g., nationalism) descriptively combine the two in the very definition of the variable (for a more detailed discussion of differences and similarities between the concepts, see Golec de Zavala, 2011, 2018; Golec de Zavala and Schatz, 2013; Golec de Zavala et al., 2019a).

2 The same dataset was used to test a different hypothesis. Different variables were assessed in this study, apart from collective narcissism (Golec de Zavala and Federico, in preparation). Variables are described in Supplementary Material.

3 https://panelariadna.pl/

4 Using OpenMx (Boker et al., 2019) in R we ran a simulation on 1000 random parameters values with 500 replication within each setup. The population model parameters where drawn from a normal distribution following smallest observed effects for all the regression paths. Covariance values between Collective narcissism and In-group satisfaction where drawn from a uniform distribution in a range of 0.6 to 0.8 Additionally each response item was set to have a random residual error with a mean of 0.5 and a standard deviation 0.25. Simulation program was set so that within each of the 1000 models with randomly generated parameters at every run, 500 simulations at varying sample size where completed. Supplementary Figure S2 plots results for each generated model filtered out for sample sizes smaller than 10 and bigger than 300. We aimed for 80% power of discovering two indirect effects and according to our results the smallest estimated sample size was *N* = 155 (power = 0.80, 95%CI[0.78; 0.86]). For a more conservative estimate we looked lower confidence interval power and concluded an optimal sample size was *N* = 190 (power = 0.86, 95%CI[0.81; 0.90]) (see Supplementary Material for details).
