# Supplementary material for: Collective Narcissism and In-Group Satisfaction Predict Opposite Attitudes Toward Refugees via Attribution of Hostility
Source: Front Psychol. 2019 Sep 4;10:1901. doi: 10.3389/fpsyg.2019.01901 (PMC6737048; doi:10.3389/fpsyg.2019.01901)
Supplement: Supplementary file 1 [file Table_1.DOCX]

Supplementary Material

# Sample size simulation for discovering an indirect effect with 80% power

Using OpenMx (Boker et al., 2019) in R we ran a simulation on 1000 random parameters values with 500 replication within each setup. The population model parameters where drawn from a normal distribution following smallest observed effects for all the regression paths. Covariance values between Collective narcissism and In-group satisfaction where drawn from a uniform distribution in a range of 0.6 to 0.8 Additionally each response item was set to have a random residual error with a mean of 0.5 and a standard deviation 0.25.

Simulation program was set so that within each of the 1000 models with randomly generated parameters at every run, 500 simulations at varying sample size where completed. Figure 2. plots results for each generated model filtered out for sample sizes smaller than 10 and bigger than 300.

We aimed for 80% power of discovering two indirect effects and according to our results the smallest estimated sample size was *N*=155 (power = .80, 95%CI[.78; .86]). For a more conservative estimate we looked lower confidence interval power and concluded an optimal sample size was *N*=190 (power = .86, 95%*CI*[.81; .90]).

**2 Other variables assessed in Study 1 (N=1066)**

***Moral Foundations*** were measured by the Polish version of the Moral Foundations Questionnaire, consisting of the full Moral Foundations Questionnaire (Graham et al., 2009): group loyalty (α =.73, *M* = 3.87, *SD* = 0.77), authority (α =.68, *M* = 3.73, *SD* = 0.77), purity (α =.79, *M* = 4.21, *SD* = 0.82), care (α =.81, *M* = 4.38, *SD* = 0.88) and fairness (α =.74, *M* = 4.26, *SD* = 0.79).

***Ideology*** was measured using a single item: “How would you describe your political orientation?” Responses included “left” (1), “center left” (2), “center” (3), “center-right” (4), “right” (5), and “It is difficult to say” (6). For the purposes of analysis, “difficult to say” responses were recoded into the center category (3), given the high number of individuals choosing this option. High scores indicated a more right-wing position (*M* = 3.09, *SD* = 1.10, for the recoded scale)

# Supplementary Tables

**Supplementary Table 1.** *Descriptive statistics for raw item scores in Study 1 (N=1066 )and Study 2 (N=419)*

| **Variables** | | **N** | **Mean** | **SD** | **Skew** | **Kurtosis** | **SE** |
| --- | --- | --- | --- | --- | --- | --- | --- |
| **Collective narcissism** | | | | | | | |
| cn1 | 1 | 1066 | 3.68 | 1.53 | -0.30 | -0.92 | 0.05 |
| cn2 | 2 | 1066 | 3.34 | 1.54 | -0.03 | -1.02 | 0.05 |
| cn3 | 3 | 1066 | 4.03 | 1.45 | -0.49 | -0.59 | 0.04 |
| cn4 | 4 | 1066 | 3.90 | 1.42 | -0.45 | -0.63 | 0.04 |
| cn5 | 5 | 1066 | 3.49 | 1.47 | -0.19 | -0.86 | 0.04 |
| **In-group satisfaction** | | | | | | | |
| is1 | 6 | 1066 | 4.51 | 1.28 | -0.90 | 0.40 | 0.04 |
| is2 | 7 | 1066 | 4.45 | 1.26 | -0.85 | 0.33 | 0.04 |
| is3 | 8 | 1066 | 4.55 | 1.22 | -0.99 | 0.77 | 0.04 |
| is4 | 9 | 1066 | 4.59 | 1.27 | -0.94 | 0.53 | 0.04 |
| **Hostile attribution bias** | | | | | | | |
| hab1 | 10 | 1066 | 3.73 | 1.60 | -0.19 | -1.06 | 0.05 |
| hab2 | 11 | 1066 | 3.52 | 1.50 | 0.04 | -0.98 | 0.05 |
| hab3 | 12 | 1066 | 3.80 | 1.47 | -0.18 | -0.85 | 0.04 |
| hab4 | 13 | 1066 | 3.79 | 1.55 | -0.25 | -0.98 | 0.05 |
| **Hostility towards refugees** | | | | | | | |
| htr1 | 14 | 1066 | 3.92 | 1.48 | -0.02 | -1.07 | 0.05 |
| htr2 | 15 | 1066 | 3.82 | 1.57 | -0.04 | -1.14 | 0.05 |
| **Variables** | | **N** | **Mean** | **SD** | **Skew** | **Kurtosis** | **SE** |
| **Collective narcissism** | | | | | | | |
| cn1 | 1 | 419 | 3.60 | 1.37 | -0.06 | -0.63 | 0.07 |
| cn2 | 2 | 419 | 3.22 | 1.31 | 0.21 | -0.32 | 0.06 |
| cn3 | 3 | 419 | 3.96 | 1.28 | -0.31 | -0.34 | 0.06 |
| cn4 | 4 | 419 | 3.81 | 1.23 | -0.15 | -0.28 | 0.06 |
| cn5 | 5 | 419 | 3.48 | 1.35 | -0.14 | -0.48 | 0.07 |
| **In-group satisfaction** | | | | | | | |
| is1 | 6 | 419 | 4.40 | 1.15 | -0.52 | 0.25 | 0.06 |
| is2 | 7 | 419 | 4.45 | 1.12 | -0.59 | 0.65 | 0.05 |
| is3 | 8 | 419 | 4.54 | 1.10 | -0.71 | 0.91 | 0.05 |
| is4 | 9 | 419 | 4.54 | 1.17 | -0.67 | 0.36 | 0.06 |
| **Hostile attribution bias** | | | | | | | |
| hab1 | 10 | 419 | 3.82 | 1.41 | -0.09 | -0.78 | 0.07 |
| hab2 | 11 | 419 | 3.45 | 1.24 | 0.29 | -0.30 | 0.06 |
| hab3 | 12 | 419 | 3.88 | 1.33 | 0.05 | -0.84 | 0.07 |
| hab4 | 13 | 419 | 3.92 | 1.33 | -0.03 | -0.75 | 0.07 |
| hab5 | 14 | 419 | 3.49 | 1.26 | 0.10 | -0.35 | 0.06 |
| **Hostility towards refugees** | | | | | | | |
| htr1 | 15 | 419 | 2.56 | 1.60 | 0.62 | -0.80 | 0.08 |
| htr2 | 16 | 419 | 3.43 | 1.78 | -0.04 | -1.34 | 0.09 |
| htr3 | 17 | 419 | 1.77 | 1.33 | 1.79 | 2.27 | 0.07 |
| htr4 | 18 | 419 | 1.83 | 1.40 | 1.66 | 1.65 | 0.07 |
| htr5 | 19 | 419 | 3.61 | 1.61 | -0.13 | -1.05 | 0.08 |
| htr6 | 20 | 419 | 2.02 | 1.46 | 1.30 | 0.54 | 0.07 |
| htr7 | 21 | 419 | 1.82 | 1.33 | 1.59 | 1.53 | 0.07 |
| htr8 | 22 | 419 | 1.90 | 1.41 | 1.47 | 1.05 | 0.07 |

**Supplementary Table 2.** *Standardized and unstandardized latent factors coefficients, Study 1 (N=1066)*

| **Latent factor** | **Indicator** | **B** | **SE** | **Z** | **p-value** | **Beta** |
| --- | --- | --- | --- | --- | --- | --- |
| **Collective narcissism** | | | | | | |
| CN | cn1 | 1.29 | 0.03 | 41.80 | <0.001 | 0.84 |
| CN | cn2 | 1.25 | 0.03 | 36.79 | <0.001 | 0.81 |
| CN | cn3 | 1.17 | 0.04 | 32.16 | <0.001 | 0.81 |
| CN | cn4 | 1.17 | 0.03 | 33.43 | <0.001 | 0.82 |
| CN | cn5 | 1.20 | 0.03 | 35.35 | <0.001 | 0.82 |
| **In-group satisfaction** | | | | | | |
| IS | is1 | 1.17 | 0.03 | 34.31 | <0.001 | 0.91 |
| IS | is2 | 1.14 | 0.04 | 32.15 | <0.001 | 0.90 |
| IS | is3 | 0.97 | 0.04 | 25.33 | <0.001 | 0.80 |
| IS | is4 | 1.15 | 0.03 | 33.91 | <0.001 | 0.91 |
| **Hostile attribution bias** | | | | | | |
| HAB | hab1 | 1.36 | 0.04 | 37.76 | <0.001 | 0.85 |
| HAB | hab2 | 1.23 | 0.03 | 35.88 | <0.001 | 0.82 |
| HAB | hab3 | 1.27 | 0.03 | 38.20 | <0.001 | 0.86 |
| HAB | hab4 | 1.36 | 0.03 | 41.28 | <0.001 | 0.88 |
| **Hostility towards refugees** | | | | | | |
| HTR | htr1 | 1.27 | 0.04 | 31.54 | <0.001 | 0.86 |
| HTR | htr2 | 1.23 | 0.04 | 27.49 | <0.001 | 0.78 |

*Note.* CN – Collective narcissism; IS – In-group satisfaction; HAB – Hostile attribution bias; HTR – Hostility towards refugees.

**Supplementary Table 3.** *Standardized and unstandardized latent factors coefficients, Study 2 (N=419)*

| **Latent factor** | **Indicator** | **B** | **SE** | **Z** | **p-value** | **Beta** |
| --- | --- | --- | --- | --- | --- | --- |
| **Collective narcissism** | | | | | | |
| CN | cn1 | 1.13 | 0.05 | 22.52 | <0.001 | 0.83 |
| CN | cn2 | 1.07 | 0.05 | 20.21 | <0.001 | 0.82 |
| CN | cn3 | 1.06 | 0.05 | 22.15 | <0.001 | 0.83 |
| CN | cn4 | 0.97 | 0.05 | 17.80 | <0.001 | 0.79 |
| CN | cn5 | 1.14 | 0.05 | 23.80 | <0.001 | 0.85 |
| **In-group satisfaction** | | | | | | |
| IS | is1 | 1.04 | 0.05 | 21.60 | <0.001 | 0.90 |
| IS | is2 | 1.04 | 0.05 | 20.80 | <0.001 | 0.93 |
| IS | is3 | 0.83 | 0.06 | 13.48 | <0.001 | 0.79 |
| IS | is4 | 1.08 | 0.05 | 22.26 | <0.001 | 0.92 |
| **Hostile attribution bias** | | | | | | |
| HAB | hab1 | 1.32 | 0.04 | 31.22 | <0.001 | 0.94 |
| HAB | hab2 | 1.02 | 0.05 | 19.94 | <0.001 | 0.82 |
| HAB | hab3 | 1.22 | 0.04 | 30.36 | <0.001 | 0.92 |
| HAB | hab4 | 1.27 | 0.04 | 32.47 | <0.001 | 0.96 |
| HAB | hab5 | 0.47 | 0.07 | 6.28 | <0.001 | 0.37 |
| **Hostility towards refugees** | | | | | | |
| HTR | htr1 | 0.78 | 0.08 | 9.46 | <0.001 | 0.49 |
| HTR | htr2 | 0.58 | 0.08 | 7.15 | <0.001 | 0.33 |
| HTR | htr3 | 1.18 | 0.07 | 16.07 | <0.001 | 0.89 |
| HTR | htr4 | 1.27 | 0.07 | 17.45 | <0.001 | 0.91 |
| HTR | htr5 | 0.60 | 0.07 | 8.45 | <0.001 | 0.37 |
| HTR | htr6 | 1.16 | 0.07 | 17.00 | <0.001 | 0.80 |
| HTR | htr7 | 1.15 | 0.07 | 17.03 | <0.001 | 0.86 |
| HTR | htr8 | 1.26 | 0.07 | 19.41 | <0.001 | 0.90 |

*Note.* CN – Collective narcissism; IS – In-group satisfaction; HAB – Hostile attribution bias; HTR – Hostility towards refugees.

**Supplementary Table 4.** *Correlations among all four latent factors for Studies 1-2*

| *Study 1 (N=1066)* | | | | |
| --- | --- | --- | --- | --- |
| **Factor 1** | **Factor 2** | **Correlation** | **SE** | **p-value** |
| CN | IS | 0.74 | 0.02 | <0.001 |
| CN | HAB | 0.54 | 0.03 | <0.001 |
| CN | HTR | 0.26 | 0.04 | <0.001 |
| IS | HAB | 0.29 | 0.04 | <0.001 |
| IS | HTR | 0.12 | 0.04 | 0.004 |
| HAB | HTR | 0.68 | 0.03 | <0.001 |
| *Study 2 (N=419)* | | | | |
| **Factor 1** | **Factor 2** | **Correlation** | **SE** | **p-value** |
| CN | IS | 0.69 | 0.04 | <0.001 |
| CN | HAB | 0.41 | 0.05 | <0.001 |
| CN | HTR | 0.13 | 0.05 | 0.008 |
| IS | HAB | 0.09 | 0.06 | 0.122 |
| IS | HTR | -0.1 | 0.05 | 0.065 |
| HAB | HTR | 0.33 | 0.04 | <0.001 |

*Note.* CN – Collective narcissism; IS – In-group satisfaction; HAB – Hostile attribution bias; HTR – Hostility towards refugees.

**Supplementary Table 5.** *Standardized parameters for alternative model 1 for Studies 1-2*

|  |  |  |  | *Study 1 (N=1066)* |  |  |  |
| --- | --- | --- | --- | --- | --- | --- | --- |
| **lhs** | **op** | **rhs** | **label** | **Beta** | **SE** | **Z** | **p-value** |
| **Regressions** | | | | | | | |
| HAB | ~ | CN | c1 | 0.53 | 0.05 | 11.66 | <0.001 |
| HAB | ~ | IS | c2 | -0.17 | 0.05 | -3.75 | <0.001 |
| HTR | ~ | CN | a1 | 0.38 | 0.06 | 6.52 | <0.001 |
| HTR | ~ | IS | a2 | -0.18 | 0.06 | -2.81 | 0.005 |
| HAB | ~ | HTR | b | 0.6 | 0.04 | 14.10 | <0.001 |
| **Mediation Effects** | | | | | | | |
| Direct_CN | := | c1 |  | 0.53 | 0.05 | 11.66 | <0.001 |
| Indirect_CN | := | a1*b |  | 0.23 | 0.04 | 6.01 | <0.001 |
| Total_CN | := | c1+(a1*b) |  | 0.75 | 0.05 | 14.61 | <0.001 |
| Direct_IS | := | c2 |  | -0.15 | 0.05 | -3.75 | <0.001 |
| Indirect_IS | := | a2*b |  | -0.11 | 0.04 | -2.82 | 0.01 |
| Total_IS | := | c2+(a2*b) |  | -0.28 | 0.06 | -4.92 | <0.001 |
|  |  |  |  | *Study 2 (N=419)* |  |  |  |
| **lhs** | **op** | **rhs** | **label** | **Beta** | **SE** | **Z** | **p-value** |
| **Regressions** | | | | | | | |
| HAB | ~ | CN | c1 | 0.69 | 0.09 | 7.46 | <0.001 |
| HAB | ~ | IS | c2 | -0.37 | 0.10 | -3.69 | <0.001 |
| HTR | ~ | CN | a1 | 0.26 | 0.07 | 4.04 | <0.001 |
| HTR | ~ | IS | a2 | -0.27 | 0.07 | -3.85 | <0.001 |
| HAB | ~ | HTR | b | 0.38 | 0.10 | 3.67 | <0.001 |
| **Mediation Effects** | | | | | | | |
| Direct_CN | := | c1 |  | 0.69 | 0.09 | 7.46 | <0.001 |
| Indirect_CN | := | a1*b |  | 0.10 | 0.02 | 4.17 | <0.001 |
| Total_CN | := | c1+(a1*b) |  | 0.79 | 0.09 | 8.74 | <0.001 |
| Direct_IS | := | c2 |  | -0.37 | 0.10 | -3.69 | <0.001 |
| Indirect_IS | := | a2*b |  | -0.10 | 0.03 | -4.01 | <0.001 |
| Total_IS | := | c2+(a2*b) |  | -0.48 | 0.10 | -4.86 | <0.001 |

*Note.* CN – Collective narcissism; IS – In-group satisfaction; HAB – Hostile attribution bias; HTR – Hostility towards refugees.

**Supplementary Table 6.** *Standardized parameters for alternative model 2 for Studies 1-2*

|  |  | *Study 1  (N=1066)* | | | |  |  |  | |
| --- | --- | --- | --- | --- | --- | --- | --- | --- | --- |
| **lhs** | **op** | **rhs** | **label** | **Beta** | **SE** | **Z** | **p-value** |  |  |
| **Regressions** | | | | | | | | |  |
| HAB | ~ | HTR | a1 | 0.72 | 0.05 | 15.58 | <0.001 |  |  |
| CN | ~ | HAB | b1 | 0.63 | 0.05 | 12.96 | <0.001 |  |  |
| CN | ~ | HTR | c1 | -0.20 | 0.05 | -3.63 | <0.001 |  |  |
| IS | ~ | HAB | b2 | 0.33 | 0.05 | 6.69 | <0.001 |  |  |
| IS | ~ | HTR | c2 | -0.13 | 0.05 | -2.51 | 0.01 |  |  |
| **Mediation Effects** | | | | | | | | | |
| Direct_CN | := | c1 |  | -0.20 | 0.05 | -3.63 | <0.001 |  |  |
| Indirect_CN | := | a1*b1 |  | 0.46 | 0.05 | 9.15 | <0.001 |  |  |
| Total_CN | := | c1+(a1*b1) |  | 0.26 | 0.04 | 6.29 | <0.001 |  |  |
| Direct_IS | := | c2 |  | -0.13 | 0.05 | -2.51 | 0.01 |  |  |
| Indirect_IS | := | a1*b2 |  | 0.24 | 0.04 | 6.24 | <0.001 |  |  |
| Total_IS | := | c2+(a1*b2) |  | 0.11 | 0.04 | 2.99 | 0.00 |  |  |
|  | *Study 2   (N=419)* | | | | | | |  |  |
| **lhs** | **op** | **rhs** | **label** | **Beta** | **SE** | **Z** | **p-value** |  |  |
| **Regressions** | | | | | | | |  |  |
| HAB | ~ | HTR | a1 | 0.56 | 0.10 | 5.63 | <0.001 |  |  |
| CN | ~ | HAB | b1 | 0.35 | 0.05 | 7.37 | <0.001 |  |  |
| CN | ~ | HTR | c1 | -0.01 | 0.07 | -0.10 | 0.92 |  |  |
| IS | ~ | HAB | b2 | 0.11 | 0.05 | 2.25 | 0.03 |  |  |
| IS | ~ | HTR | c2 | -0.19 | 0.07 | -2.64 | 0.01 |  |  |
| **Mediation Effects** | | | | | | | |  |  |
| Direct_CN | := | c1 |  | -0.01 | 0.07 | -0.10 | 0.92 |  |  |
| Indirect_CN | := | a1*b1 |  | 0.20 | 0.04 | 4.46 | <0.001 |  |  |
| Total_CN | := | c1+(a1*b1) |  | 0.19 | 0.07 | 2.59 | 0.01 |  |  |
| Direct_IS | := | c2 |  | -0.19 | 0.07 | -2.64 | 0.01 |  |  |
| Indirect_IS | := | a1*b2 |  | 0.06 | 0.03 | 2.08 | 0.04 |  |  |
| Total_IS | := | c2+(a1*b2) |  | -0.13 | 0.07 | -1.88 | 0.06 |  |  |

*Note.* CN – Collective narcissism; IS – In-group satisfaction; HAB – Hostile attribution bias; HTR – Hostility towards refugees.

## Supplementary Figures


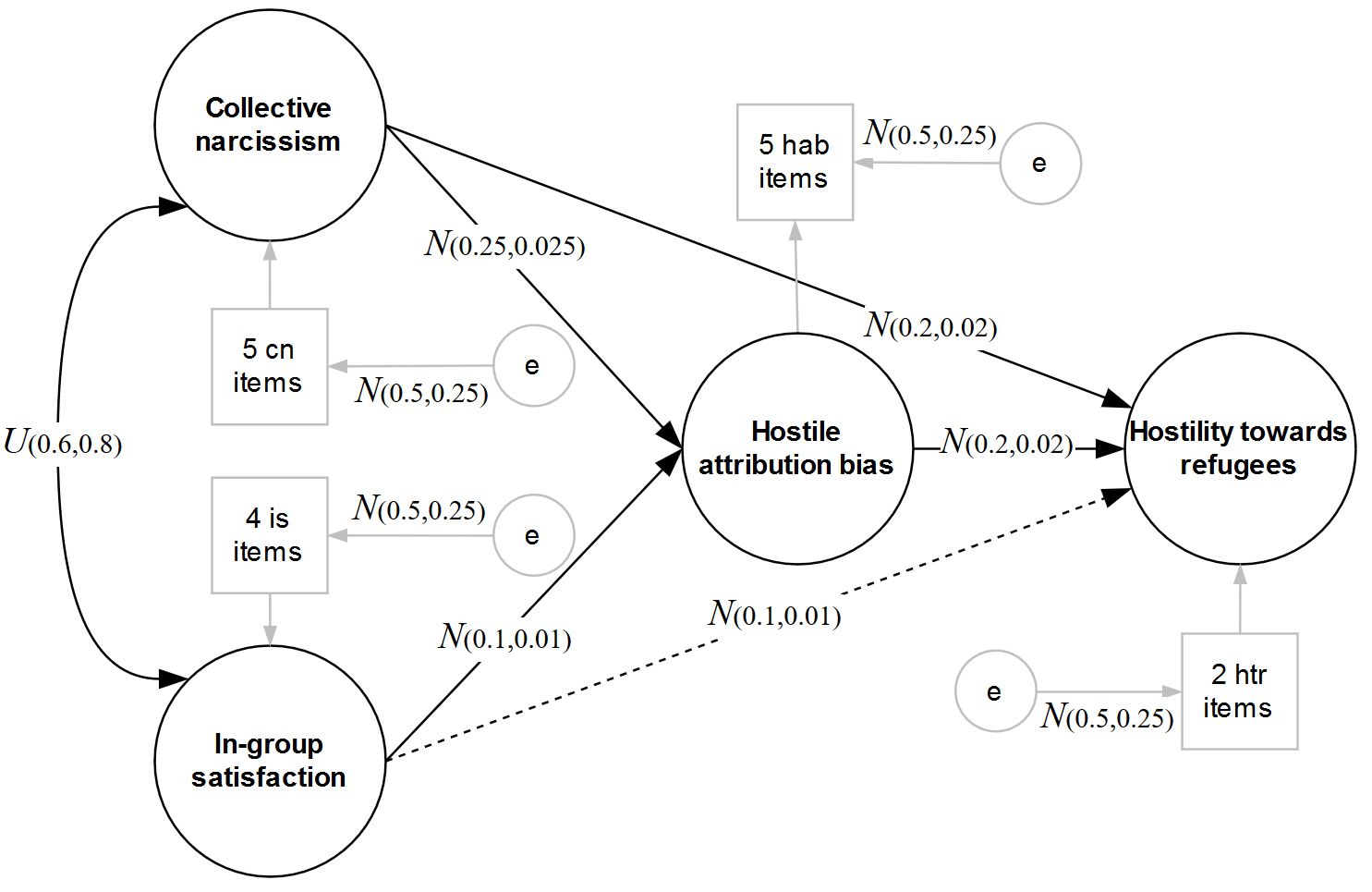


**Supplementary Figure 1***. Data generation model.*

**
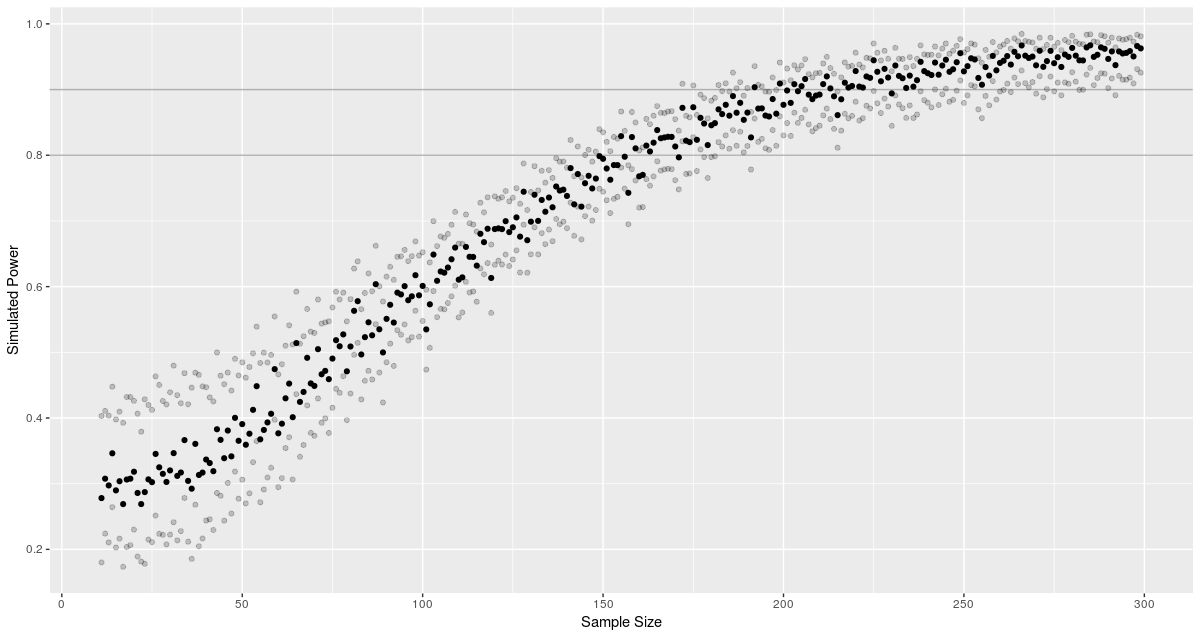
**

**Supplementary Figure 2.** *Power to discover an indirect effect based on varying sample size and model parameters Monte Carlo simulation.*
