# Supplementary material for: Collective Narcissism and In-Group Satisfaction Predict Opposite Attitudes Toward Refugees via Attribution of Hostility
Source: Front Psychol. 2019 Sep 4;10:1901. doi: 10.3389/fpsyg.2019.01901 (PMC6737048; doi:10.3389/fpsyg.2019.01901)
Supplement: Supplementary file 2 [file Table_2.DOCX]

**Codebook**

**Study 1**

**sex – What is your gender?**

1 – female, 2- male

**age – What is your age?**

1 – 15-17, 2 – 18-24, 3 – 25-34, 4 – 35-44, 5 – 45-54, 6 – 55 or more

**year – What is your age exactly?**

**cities – Where do you live?**

1 – countryside, 2 – small town (up to 20 000 inhabitants), 3 – medium town (20-99 000 inhabitants), 4 – big town (100 – 500 000 inhabitants), 5 - big city (more than 500 000 inhabitants)

**edu – What is your education?**

1 – secondary, 2 – technical, 3 – high school, 4 – post-secondary, 5 – bachelor, 6 – master’s degree

**q1 - If the election to Parliament took place nest Sunday would you go to vote?**

1 – definitely no, 2 – rather no, 3 – rather yes, 4 – yes, 5 – difficult to say

**q2 - Which political party would you vote for?**

1 – Prawo i Sprawiedliwość, 2 – Platforma Obywatelska, 3 – Kukiz ’15, 4 – PSL, 5 – Nowoczesna, 6 – Sojusz Lewicy Demokratycznej, 7 – Korwin, 8 – Razem, 9 – I don’t know

**Moral Foundations Questionnaire (Graham et al., 2009)**

1 – I definitely do not take it into account, 2 – I don’t take it into account, 3 – I rather don’t take it into account, 4 – I rather take it into account, 5 – I take it into account, 6 – I absolutely take it into account

harmitem1 - Whether or not someone suffered emotionally

fairitem1 - Whether or not some people were treated differently than others

groupitem1 - Whether or not someone’s action showed love for his or her country

athoritem1 - Whether or not someone showed a lack of respect for authority

pureitem1 - Whether or not someone violated standards of purity and decency

harmitem2 - Whether or not someone cared for someone weak or vulnerable

fairitem2 - Whether or not someone acted unfairly

groupitem2 - Whether or not someone did something to betray his or her group

athoritem2 - Whether or not someone conformed to the traditions of society

pureitem2 - Whether or not someone did something disgusting

harmitem3 - Whether or not someone was cruel

fairitem3 - Whether or not someone was denied his or her rights

groupitem3 - Whether or not someone showed a lack of loyalty

athroitem3 - Whether or not an action caused chaos or disorder

pureitem3 - Whether or not someone acted in a way that God would approve of

1 – absolutely disagree, 2 – disagree, 3 – rather disagree, 4 – rather agree, 5 – agree, 6 – absolutely agree

harmitem4 - Compassion for those who are suffering is the most crucial virtue

fairitem4 - When the government makes laws, the number one principle should be ensuring that everyone is treated fairly

groupitem4 - I am proud of my country’s history

athoritem4 - Respect for authority is something all children need to learn.

pureitem4 - People should not do things that are disgusting, even if no one is harmed.

harmitem5 - One of the worst things a person could do is hurt a defenseless animal

fairitem5 - Justice is the most important requirement for a society.

groupitem5 - People should be loyal to their family members, even when they have done something wrong.

athoritem5 - Men and women each have different roles to play in society

pureitem5 - I would call some acts wrong on the grounds that they are unnatural

harmitem6 - It can never be right to kill a human being

fairitem6 - I think it’s morally wrong that rich children inherit a lot of money while poor children inherit nothing

groupitem6 - It is more important to be a team player than to express oneself

athoritem6 - If I were a soldier and disagreed with my commanding officer’s orders, I would obey anyway because that is my duty.

pureitem6 - Chastity is an important and valuable virtue.

**cn - Collective Narcissism Scale (Golec de Zavala et al. 2009)**

1 – absolutely disagree, 2 – disagree, 3 – rather disagree, 4 – rather agree, 5 – agree, 6 – absolutely agree

cn1 – Poles deserve special treatment

cn2 – not many people seem to fully understand the importance of Poles

cn3 - it really makes me angry when someone criticizes Poles

cn4 - if Poles had a major say in the world, the world could be a much better place

cn5 - I will never be satisfied until Poles get the recognition they deserve

**is - In-group Satisfaction subscale of Ingroup Identification Scale (Leach et al., 2008)**

1 – absolutely disagree, 2 – disagree, 3 – rather disagree, 4 – rather agree, 5 – agree, 6 – absolutely agree

is1 - I am glad to be Pole

is2 - I think Poles have a lot to be proud of.

is3 – It is pleasant to be Pole.

is4 - Being Pole gives me a good feeling.

**control – I have control over my life.**

1 – absolutely disagree, 2 – disagree, 3 – rather disagree, 4 – rather agree, 5 – agree, 6 – absolutely agree

**esteem – I have high self-esteem.**

1 – absolutely disagree, 2 – disagree, 3 – rather disagree, 4 – rather agree, 5 – agree, 6 – absolutely agree

**narc – I am narcissistic.**

1 – absolutely disagree, 2 – disagree, 3 – rather disagree, 4 – rather agree, 5 – agree, 6 – absolutely agree

**Social Distance / Feelings**

ref1 - I have warm feelings towards Syrian refugees

ref1rev – reversed for ref1

ref2 - I would have nothing against a member of my family marrying a Syrian refugee

ref2rev – reversed for ref2

ref3 - I don’t trust Syrian refugees

ref - I would have nothing against a family of Syrian refugees moving in into a neighbouring flat/house

ref5 - I would have nothing against Syrian refugees settling in my district

ref6 – I would have nothing against Syrian refugees establishing their place of worship in my district

ref7 - I would have nothing against Syrian refugees children attending school together with my children

ref8 - Poland should accept more Syrian refugee

**Hostile attribution bias**

refhost1 - Syrian refugees threaten our national security

refhost2 - Syrian refugees are hostile towards Poles

refhost3 - Syrian refugees are aggressive

refhost4 - Syrian refugees are dangerous

refhost5 - Syrian refugees are helpless

**party - which political party represents best your political outlook?**

1 – Prawo i Sprawiedliwość, 2 – Platforma Obywatelska, 3 – Kukiz ’15, 4 – PSL, 5 – Nowoczesna, 6 – Sojusz Lewicy Demokratycznej, 7 – Korwin, 8 – Razem, 9 – nont of listed

**familysituation – What is your family financial situation?**

1 – very bad, 2 – bad, 3 – rather bad, 4 – difficult to say, 5 – rather good, 6 – good, 7 – very good

**economyPoland – How do find Polish economic situation?**

1 – very bad, 2 – bad, 3 – rather bad, 4 – difficult to say, 5 – rather good, 6 – good, 7 – very good

**libcon – What is your political orientation?**

1 - definitely conservative, 2 – conservative, 3 - rather conservative, 4 - rather liberal, 5 – liberal, 6 - definitely liberal

**leftright - how would you describe your political orientation?**

1 – leftist, 2 – center-leftist, 3 – center, 4 – center-rightist, 5 – rightist, 6 – difficult to say

**job – What is your professional status?**

1 – job agreement, 2 – job on order, 3 – contract work, 4 – own business, 5 – unemployed, 6 – retired, 7 – student

**children – Do you have children?**

1 – yes, 2 – no

**marital – What is your marital status?**

1 – single, 2 – married, 3 – informal relationship, 4 – widow, 5 – divorced

**income – What is your netto month income?**

1 – no income, 2 – up to 2000 PLN, 3 – 2001-3000 PLN, 4 – 3001-4000 PLN, 5- 4001-5000, 6 – more than 5000, 7 – I prefer not to say

**control – control questions**

control 1 – I was good at math at school.

control 2 - It is better to do good than to do bad.
