# Supplementary material for: Collective Narcissism and In-Group Satisfaction Predict Opposite Attitudes Toward Refugees via Attribution of Hostility
Source: Front Psychol. 2019 Sep 4;10:1901. doi: 10.3389/fpsyg.2019.01901 (PMC6737048; doi:10.3389/fpsyg.2019.01901)
Supplement: Supplementary file 3 [file Table_3.DOCX]

**Codebook**

**Study 2**

**sex – What is your gender?**

1 – female, 2- male

**age – What is your age?**

1 – 15-17, 2 – 18-24, 3 – 25-34, 4 – 35-44, 5 – 45-54, 6 – 55 or more

**year – What is your age exactly?**

**cities – Where do you live?**

1 – countryside, 2 – small town (up to 20 000 inhabitants), 3 – medium town (20-99 000 inhabitants), 4 – big town (100 – 500 000 inhabitants), 5 - big city (more than 500 000 inhabitants)

**edu – What is your education?**

1 – secondary, 2 – technical, 3 – high school, 4 – post-secondary, 5 – bachelor, 6 – master’s degree

**orient_pol – What is your political orientation?**

1 – definitely liberal, 2 – liberal, 3 – rather liberal, 4 – rather conservative, 5 – conservative, 6 – definitely conservative

**cn - Collective Narcissism Scale (Golec de Zavala et al., 2009)**

1 – absolutely disagree, 2 – disagree, 3 – rather disagree, 4 – rather agree, 5 – agree, 6 – absolutely agree

cn1 – Poles deserve special treatment

cn2 – not many people seem to fully understand the importance of Poles

cn3 - it really makes me angry when someone criticizes Poles

cn4 - if Poles had a major say in the world, the world could be a much better place

cn5 - I will never be satisfied until Poles get the recognition they deserve

**is - In-group Satisfaction subscale of Ingroup Identification Scale (Leach et al., 2008)**

1 – absolutely disagree, 2 – disagree, 3 – rather disagree, 4 – rather agree, 5 – agree, 6 – absolutely agree

is1 - I am glad to be Pole

is2 - I think Poles have a lot to be proud of.

is3 – It is pleasant to be Pole.

is4 - Being Pole gives me a good feeling.

**refhost – To what extent you agree or disagree Syrian refugees are:**

1 – absolutely disagree, 2 – disagree, 3 – rather disagree, 4 – rather agree, 5 – agree, 6 – absolutely agree

refhost1 - a threat to Polish national security

refhost2 – hostile towards Polish

refhost3 – aggressive

refhost4 – dangerous

refhost5 – helpless (refhost5_rev)

**p1_2_r1 – Please indicate to what extent you would like to engage in each of the following behavior with respect to the Syrian refugees:**

1 – absolutely no, 2 – no, 3 – rather no, 4 – rather yes, 5 – yes, 6 – absolutely yes

p1_2_r1– help them

(p1_2_r1_rev) – reversed for help them

p1_2_r2 – confront them

p1_2_r3 – oppose them

p1_2_r4 – hurt them

p1_2_r5 – take care of them

(p1_2_r5rev) – reversed for take care of them

p1_2_r6 – humiliate them

p1_2_r7 – leave them alone

(p1_2_r7) – reversed for leave them alone

p1_2_r8 – leave them to their fate

p1_2_r9 – intimidate them

p1_2_r10 – injure them

p1_2_r11 – don’t look at them

p1_2_r12 - offend them
